# Can We Repurpose FDA-Approved Alefacept to Diminish the HIV Reservoir?

**DOI:** 10.4172/imt.1000104

**Published:** 2015-11-30

**Authors:** Asifa Zaidi, Qinglai Meng, Daniel Popkin

**Affiliations:** Department of Dermatology, Case Western Reserve University School of Medicine, Cleveland, OH 44106, USA

**Keywords:** Alefacept, NK cells, ADCC, CD4^+^ T cells, HIV reservoir

## Abstract

Current anti-retroviral treatment (ART) for HIV is effective in maintaining HIV at undetectable levels. However, cessation of ART results in immediate and brisk rebound of viremia to high levels. This rebound is driven by an HIV reservoir mainly enriched in memory CD4^+^ T cells. In order to provide any form of functional HIV Cure, elimination of this viral reservoir has become the focus of current HIV cure strategies. Alefacept was initially developed for the treatment of chronic plaque psoriasis. Alefacept is a chimeric fusion protein consisting of the CD2-binding portion of human leukocyte function antigen-3 (LFA3) linked to the Fc region of human IgG1 (LFA3-Fc). Alefacept was designed to inhibit memory T cell activation that contributes to the chronic autoimmune disease psoriasis by blocking the CD2 coreceptor. However, it was found to deplete memory T cells that express high levels of CD2 via NK cell-mediated antibody dependent cell cytotoxicity (ADCC) *in vivo*. Phase II and phase III clinical trials of alefacept with psoriasis patients demonstrated promising results and an excellent safety profile. Subsequently, alefacept has been successfully repurposed for other memory T cell-mediated autoimmune diseases including skin diseases other than psoriasis, organ transplantation and type I diabetes (T1D). Herein, we review our specific strategy to repurpose the FDA approved biologic alefacept to decrease and hopefully someday eliminate the HIV reservoir, for which CD2^hi^ memory CD4^+^ T cells are a significant contributor.

## Introduction

**Figure 1:**
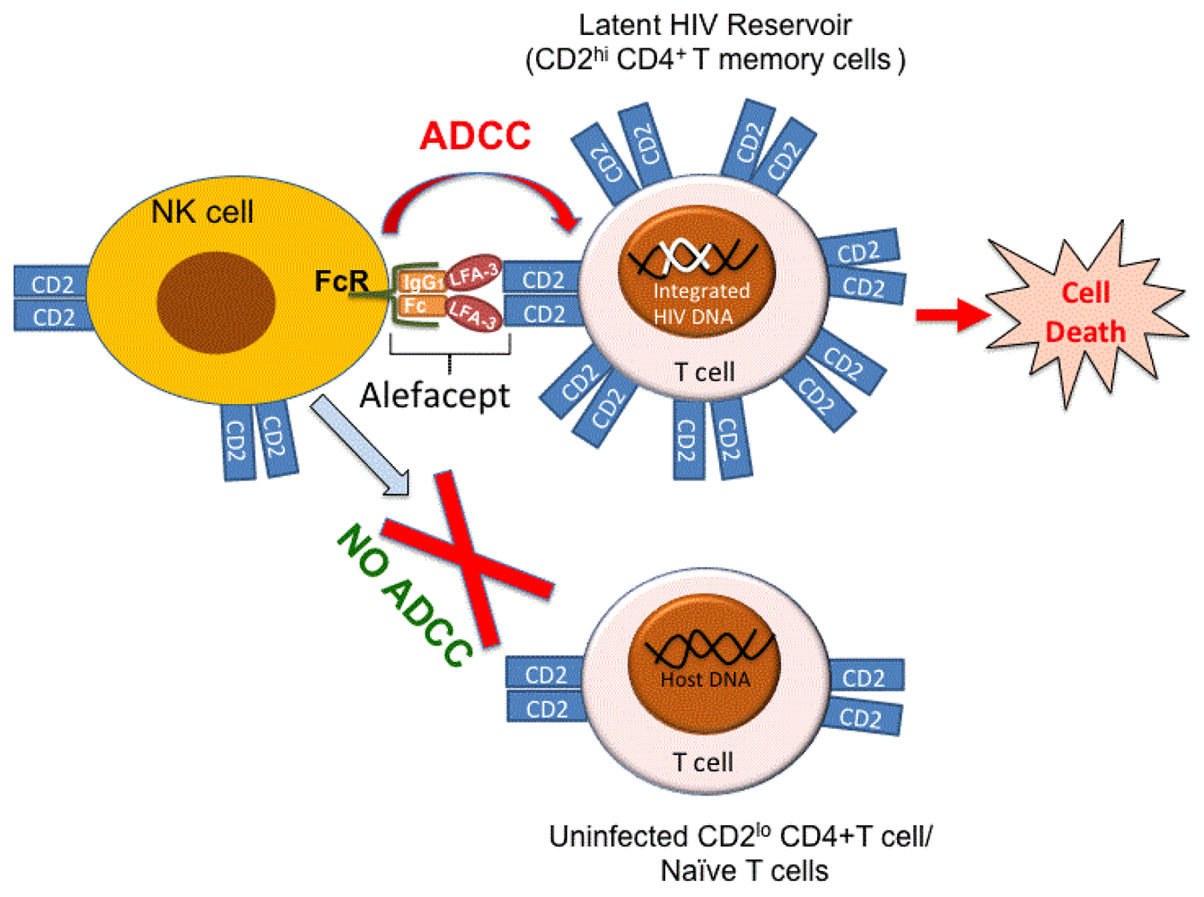
Alefacept-mediated selective killing of memory CD2^hi^ CD4^+^ T cells harboring HIV genome via engagement of CD2 on T cells and CD16 (FcR) on NK cells but not CD2^lo^ CD4^+^ T cells (Uninfected or naïve cells). ADCC: Antibody Dependent Cellular Cytotoxicity; FcR: Fc Receptor (CD16); LFA-3: Lymphocyte Function-Associated Antigen 3

Alefacept was FDA-approved in 2002 for the treatment of chronic plaque psoriasis. Psoriasis is a well-established effector memory T cell-mediated autoimmune disease, where, memory CD4^+^CD45RO^+^and CD8^+^CD45RO^+^ T cells mediate hyperproliferation of keratinocytes that results in typical psoriasis symptoms [1–3]. Alefacept is a chimeric protein created by fusion of the first domain of lymphocyte function-associated antigen 3 (LFA-3) that binds to CD2 and Fc region (the CH_2_ and CH_3_ hinge region) of human IgG1 (LFA3-Fc). Alefacept was designed to inhibit T cell activation by blocking the interaction between CD2 (expressed on T cells) and LFA-3 (expressed on antigen presenting cells) [4,5]. However, later studies with human PBMCs, transgenic mice expressing human CD2 and engineered alefacept (that have amino acid substitutions in Fc region that impact Fc gamma receptor binding) showed that alefacept eliminates CD2^hi^ memory T cells via NK-mediated antibody-dependent cell-cytotoxicity (ADCC) [6,7]. Activated memory T cells express higher levels of CD2 than naïve T cells [8,9] that result in preferential binding of alefacept and selective removal of activated memory T cells [5,7] (Figure-1). Hence, alefacept not only blocks T cell activation and proliferation by binding to CD2 and blocking LFA3/CD2 engagement on T cells but also engages CD16 on NK cells for ADCC to deplete CD2^hi^ expressing memory T cells.

Alefacept is effective for psoriasis and other T cell-mediated autoimmune diseases [5,10–18]. Alefacept responses are noteworthy for their durability. Psoriatic responders can have extended treatment-free and disease-free periods, which lessen the need for treatment over time. In clinical trials involving more than thousand patients, single course (12 once-weekly injections of 7.5 mg of alefacept intravenously [15] or 15 mg intramuscular [19] followed by a 12-week treatment-free period) achieved significant and preferential reduction in absolute counts of CD4^+^ and CD8^+^ memory T-cell subsets (approximately 20–75% maximum reduction) but not the naïve T cell population in circulation from baseline (5,10,13–18). There was a greater reduction in CD8^+^ memory cells compared to the CD4^+^ population and this difference may be due to differential expression of CD2 on their cell surface. Further efficacy of alefacept has also been seen with each subsequent course of treatment. The median remission period was 7–8 months in patients who responded to alefacept. In addition, alefacept significantly improved patients’ quality of life and was well tolerated, with no reports of disease rebound on completion of therapy, organ toxicity, opportunistic infections, and no significant immunogenicity. Alefacept has been well tolerated in children with no reports of drug-associated serious adverse events and no between-group (alefacept vs. placebo) differences in overall rates of adverse events except chills.

After success in psoriasis, alefacept has been used in treatment of other autoimmune diseases. Some of the studies using alefacept for other autoimmune diseases have shown a beneficial effect. We will discuss briefly both successes and failures and emphasize insights obtained from these clinical trials.

## Repurposing of Alefacept in Different Human Diseases: Successes and Failures

Alefacept has not only been used for the treatment of psoriasis but was successfully repurposed for other diseases that involved T cell activation such as type I diabetes (T1D) [20,21], preconditioning in allogeneic hematopoietic stem cell transplantation (HSCT) of pediatric patients with life-threatening nonmalignant diseases [22], kidney and liver transplantation [23–25], atopic dermatitis (AD) [26,27], nephrogenic systemic fibrosis (NSF) [28], cutaneous sarcoidosis [29] and palmar plantar pustulosis [30].

### Successes

#### Type 1 diabetes (T1D)

T1D is a chronic autoimmune disease that leads to progressive destruction of pancreatic beta cells by self-reactive memory CD4+ and CD8+ T cells [31,32]. These autoreactive T cells are comparatively resistant to suppression by regulatory T cells (Tregs) or downregulation by immune-modulating agents. Further emphasis for a role of autoreactive T cells in T1D pathogenesis is supported by case studies of T1D patients where reoccurrence of pancreatic beta cell destruction is observed after pancreatic grafts from HLA-identical twins or siblings [33–35]. This phenomenon of selective destruction of beta cells was also observed in recipients of cadaveric pancreatic grafts [36] and in recipients of islet allografts [37] without evidence of alloimmune rejection [34–36]. Further studies strongly suggested that recurrence of T1D autoimmunity was due to autoreactive, antigen-specific memory CD8^+^ and CD4^+^ T cells after islet and pancreas transplantation [37–40]. In a multicenter, randomized, double-blind, placebo-controlled clinical trial of recent onset T1D patients, treatment with alefacept, preserved their C-peptide secretion, reduced insulin use and hypoglycemic events, and induced favorable immunologic profiles at one year, even after one year of termination of therapy. As expected, alefacept treatment in this study, significantly depleted CD4^+^ and CD8^+^ central memory T cells (Tcm) and effector memory T cells (Tem), preserved Tregs, increased the ratios of Treg to Tem and Tcm, and increased the percentage of PD-1 expressing CD4^+^ Tem and Tcm [20,21]. Preservation of Tregs, was the major finding in the T1DAL trial, and was plausibly due to the lower expression of CD2 on Tregs compared to the higher levels on memory T cells. This observation had not been investigated earlier in psoriasis trials.

#### Organ transplantation

Allogeneic hematopoietic stem cells: Alloimmunization, a T cell-mediated response, poses a major hurdle to the engraftment of allogeneic tissues in patients. Several current studies support alefacept-mediated depletion of CD2^hi^ T cells in patients during allogeneic hematopoietic stem cell transplant, liver or kidney [22–25]. All patients developed post-transplant graft-versus-host disease (GVHD) but GVHD resolved even after termination of alefacept therapy and patients did not require immunosuppressive drugs [22]. A case series involving three pediatric patients treated with a short course (5 doses/week) of high dose (0.25 or 0.5 mg/kg/dose) alefacept demonstrated sustained, full donor engraftment of allogeneic hematopoietic stem cells in spite of their greater risk for graft rejection because of their non-malignant diseases (NMDs) and their extensive transfusion history, the use of mismatched donors, and the use of a reduced-intensity conditioning regimen. After five weekly doses of alefacept, selective loss of natural killer (NK) cells was observed, in addition to CD2^hi^ memory T cells. Furthermore, these three patients had successful full-donor engraftment by day 100, were able to be withdrawn from immune suppressive agents and they sustained stable full-donor engraftment even after more than two years post-transplantation. These results indicate that high dose alefacept is feasible and safe in heavily transfused patients and depletes both memory T cells and NK cells [22]. Similarly, based on a total response rate of 55% in a phase II trial of BTI-322 (antibody directed to CD2 antigen) for steroid-refractory acute GVHD [41], a standard dose of alefacept (15 mg intramuscular) was used for treatment of seven patients in eight acute GVHD episodes of skin, GI and liver with grade 2–4 [25]. There was an initial rapid response (within days) to alefacept in all patients even those with grade 4 GVHD involving skin (more than 75% body surface area) that was not responding to any prior treatments. Complete remission occurred only in one out of four patients with initial response in 40 days. There were no acute alefacept-related side effects. However, treatable infection with aspergillus sinusitis, pneumonia, bacteremia, pharyngeal thrush, pancytopenia and hemorrhagic cystitis were reported as late side effects. In addition, CMV reactivation was observed in three patients on alefacept. All these studies were limited in size (n=3 or 7) but showed a beneficial effect of alefacept and need to be confirmed with further investigations studying larger patient cohorts.

##### Renal transplant

Alefacept has also shown promising results in preclinical studies of cardiac and renal allograft survival in non-human primates and also in combination with CTLA4-Ig and sirolimus [42,43]. However, alefacept in combination with belatacept and sirolimus demonstrated negative results [44]. Hence, these studies suggest that alefacept success may be specific to unique combination therapy regimens.

##### Postorthotopic liver transplantation (OLT)

Similarly, alefacept positive response was reported in a 60-year-old man with cirrhosis status-OLT, who was not responding to other therapies. This patients’ condition was deteriorating despite high-dose steroids, filgrastim support, and rabbit antithymocyte globulin, therefore off-label use of the immunosuppressant alefacept was recommended. After the initial 30 mg alefacept dose, the patients’ blood counts improved and remained stable 13 months later [24]. This patient had GVHD after OLT confirmed by short tandem repeat (STR) analysis of four samples; a buccal swab (recipient DNA), donor DNA–Allogen labs, peripheral blood and T-cell enriched fraction from peripheral blood.

##### Skin diseases other than psoriasis

Other report alefacept successes include, improvement or stabilization of cutaneous disease of three patients with nephrogenic systemic fibrosis [28], a 46-year-old patient with recalcitrant lupus pernio (sarcoidosis) [29] and improvement in 15 palmar plantar pustulosis patients that included one patient with remission [30]. Additionally, two patients with vitiligo coexisting and colocalizing with psoriasis responded after alefacept treatment [45].

Of note, except for psoriasis clinical trials (>3000 subjects), these studies had very small patient numbers. Repurposing efforts for alefacept have been strongest for a T1D indication at present.

### Failures

Alefacept clinical trials data for 2, indications collected over a three-year period demonstrated favorable safety but little efficacy for renal transplant and vitiligo.

#### Renal transplant

In a phase II clinical trial with 218 adult patients of *de novo* renal transplant recipients, alefacept in combination with standard triple immunosuppressive regimen of tacrolimus, mycophenolate mofetil (MMF) and corticosteroids has shown no clear efficacy benefit despite the successful depletion of memory T cells by alefacept. In this study, no survival benefit or rejection rate improvement was observed and malignancies were increased in the alefacept treatment group [23,46].

#### Vitiligo

Similarly, no benefit in vitiligo after alefacept treatment was reported. Four adult patients with widespread vitiligo (acquired autoimmune pigment destruction, affecting a body surface area of ≥ 5%) received alefacept treatment. Repigmentation was observed in only one patient. All patients tolerated alefacept without any adverse events [47].

These studies indicate that alefacept can provide benefit in T cell-mediated autoimmune diseases via depletion of memory T cells.

## Challenges in HIV Cure: The HIV Latent Reservoir

Memory CD4^+^ T cells are the best-characterized latent reservoir for HIV, which remains the major obstacle for an HIV cure. The “reservoir” for HIV infection is defined as a population of cells in which HIV persists despite anti-retroviral therapy (ART) [48]. ART reduces plasma viremia below detection (≤ 50 copies/ml) limits by preventing viral replication via targeting viral entry, reverse transcription of the viral genome, integration into the genome of the host cell and maturation of viral proteins. Therefore, ART is very effective in controlling HIV infection, improving quality of life, and in reducing the death rate of HIV-infected individuals. However, decay of the HIV reservoir occurs so slowly in HIV patients on ART that it is not clinically meaningful [49]. In addition, ART has no effect in controlling the establishment of the HIV latent reservoir even when ART is started as early as 2 days after infection [50–54]. Presumably secondary to this viral reservoir, cessation of ART causes viral rebound within 2 weeks and clinical progression of AIDS in most of the patients except for rare controllers (e.g., post-treatment controllers [55,56] and elite controllers) [57–59]. These observations suggest that there is a necessity to eliminate latent reservoir to achieve clinical cure of HIV and ART free lifespan. Discontinuation of ART poses additional risk of transmission of disease to others.

Furthermore, long-term toxicity, stigma and cost associated with ART, generates a great necessity of finding cure for HIV that is critical for patient care worldwide.

Apart from memory CD4^+^ T cells, HIV compartmentalization occurs both at the cellular level as well as different anatomical sites referred to as “viral sanctuaries” [60]. The CD4^+^ monocyte-macrophage cell lineage including circulating monocytes, may contribute to the HIV reservoir. These cells may play a critical role in spreading infection to the central nervous system (CNS). Moreover, due to their ability to cross the blood–brain barrier they could serve a role in extra-CNS dissemination. Additional myeloid CNS cells that may contribute to the HIV reservoirs including meningeal macrophages, microglia and perivascular cells [61,62]. Notably, the viral infection is these cells are non-cytopathic. In addition to CD4^+^ blood cells, the anatomical sites that serves as viral sanctuaries during ART include lymph nodes, brain, gastrointestinal tract, genital tract, semen, kidney and lungs [63,64]. Several studies suggest that some of these viral sanctuaries, due to anatomical barrier features, might restrict the penetration of antiretroviral drugs thus facilitating viral replication as well as the evolution of drug resistance [64]. It may be critical to include the strategies for elimination of these viral sanctuaries to achieve functional HIV cure. We will discuss the plausible role of alefacept on CD4^+^ HIV reservoir cells present in different viral sanctuaries that are likely most to achieve a functional HIV cure.

Currently, there are several approaches underway to eliminate the latent HIV reservoir. The “Berlin patient” case enhanced greater optimism in the field of HIV cure. After bone marrow transplantation (BMT) with HIV-resistant CCR5-deficient hematopoietic stem cells, the Berlin patient had undetectable (less than one copy/ml) HIV RNA over six years off ART [65,66]. This observation also demonstrates that HIV cure can be achieved. However, BMT-associated strategies have a high associated morbidity and mortality, are expensive and thus overall impractical as the basis for an HIV cure strategy. However, the Berlin patient has provided much insight for the development of alternative cure strategies.

Complete eradication of the HIV reservoir bears several challenges. These include restricting or blocking residual levels of viral replication during ART in anatomical compartments where an adequate concentration of drug may not be attainable, quantifying pools of cells carrying silent integrated genomes that may be reactivated, and enhancing immune surveillance that normally fails at controlling residual replication and reactivation from latently infected cells. To overcome these challenges, our lab has focused on strategies to deplete the HIV latent reservoir in memory CD4^+^ T cells by augmenting and directing the innate immune response. We have focused on memory CD4^+^ T cells as they are the best characterized and therefore measurable site for the HIV reservoir in an albeit evolving and controversial field.

The time to viral rebound correlates with total viremia during acute infection and with size of latent HIV reservoir at the time of ART discontinuation [67]. This supports that reducing the size of the reservoir may extend the viral free lifespan or time to viral rebound (aka “functional cure”), even if HIV reservoir depleting strategies fail to be complete.

## Proposal: Use of Alefacept in Reducing HIV Reservoir by NK Cell-Mediated ADCC

Memory CD4^+^ T cells that express high levels of surface CD2 antigen (the target antigen for alefacept) have been shown to be enriched for latent virus [68]. Moreover, alefacept, in phase I and II clinical trials of psoriasis patients, demonstrated specific and significant depletion of CD2^hi^ memory CD4^+^ T cells via NK cell-mediated ADCC and remission of their disease [13–15]. NK cells have also been implicated for clearance and reduction in HIV reservoir [69–73], including recent studies with IFN-α [74,75]. Success of alefacept in clinical trials of psoriasis and an excellent safety profile, provides a rationale to repurpose alefacept to reduce the HIV reservoir by depleting CD2^hi^ CD4^+^ T cells (Figure 1). In addition, alefacept has several advantages as a tool for decreasing HIV reservoir; this biologic can be used transiently to facilitate significant reduction of the CD2^hi^ CD4^+^ T cell pool with no impairment of primary or secondary antibody responses to a neoantigen or memory responses to a recall antigen [76]. Moreover, single or multiple dosing regimens can be used safely as was done in psoriasis clinical trials [19,77–79].

In summary, we hypothesize that alefacept may be an excellent candidate as part of an HIV Cure strategy based on two categories of rationale:

- Alefacept has an excellent safety profile since 2002 FDA approval
- Depletion of memory CD4^+^ T cells, secondary to CD2^hi^ surface expression is well documented after alefacept treatment and coincides with our current knowledge of the HIV reservoir

## Alefacept in Reducing HIV Reservoir: Challenges

Alefacept, in all clinical trials, demonstrated significant reduction in memory CD4^+^ T cells but spared naïve T cells. However, alefacept depletes memory CD8^+^ T cell population as well, likely due to similar CD2 surface expression. CD8^+^ T cells are critical in controlling virus-infected cells and reducing the CD8^+^ T cell count might cause unacceptable transient immunosuppression. It is unclear whether transient depletion of CD8^+^ T cells with one course of alefacept will have adverse effects in an HIV^+^ patient who is well-controlled on antiretroviral therapy. Therefore, empirically determining multiple dose regimens will be necessary, and one can draw from the psoriasis literature in which some psoriatics showed significant durable improvement after a single 12-dose cycle of alefacept while others benefited from additional treatment with alefacept to alleviate their disease.

In addition to memory CD8^+^ T cells in HIV patients, NK cells also participate in regulating virus-infected cells. However, a second major challenge is that HIV patients on ART have hypofunctional NK cells compared to normal subjects. Hypofunctional NK cell mediated ADCC by alefacept might be less efficient in HIV patients than as observed for psoriasis patients. To overcome this potential challenge high dose and short course regimens may be beneficial. High doses of alefacept in allogeneic hematopoietic stem cells transplant have demonstrated efficacy [22] and several courses of low doses of alefacept in phase III clinical trial of psoriasis have also been performed with no adverse effects observed [15].

Regulatory T cells (Tregs) are also critical for immune response maintenance. In psoriasis, Tregs frequency was minimally affected after alefacept treatment of 22 psoriasis patients despite high CD2 expression. Rigby *et al*. [80] have reported that the CD2 expression on Tregs (at baseline of subjects that were newly diagnosed with type I diabetes (T1D)) were similar to naïve T cells and lower compared to memory T cells. In addition, Rigby *et al*. [21] has also demonstrated that alefacept treatment did not alter the frequency of Tregs during the entire study period and even after 2 years of alefacept treatment in subjects diagnosed with T1D. Based on these data and data from psoriasis clinical trials, we speculate that the Tregs in HIV patients will also be preserved, if ART and/or HIV infection does not modulate the CD2 expression levels on their surface. Moreover, these data indicate that alefacept treatment is not likely to cause apoptosis in Tregs. CD2 signaling already present *in vivo* is required for survival and differentiation of Tregs [81,82]. Whether this signaling is affected by alefacept is unknown. Overall Tregs were preserved up to 2 years after treatment in studies led by Mark Rigby [80,81]. It is very difficult to predict whether Treg function or IL-10 production by Tregs will be affected by alefacept in HIV^+^ individuals. This requires experimental evidence that is not available in the literature at this moment. However, if alefacept inhibits the maturation or function of Tregs *in vivo*, this likely is beneficial in the context, of HIV infection. It has been reported that long-term non-responders and elite controllers have lower frequency of Tregs in peripheral blood and rectal mucosa compared to regular progressers [83].

Several recent studies with other biologics including antibodies and fusion proteins already used in clinics as therapeutic agents, and mouse models, demonstrated that monocytes/macrophages could induce antibody-dependent cellular phagocytosis (ADCP). Macrophages express all classes of Fcγ receptors (FcγR), in contrast to NK cells, which primarily express FcγRIIIa. The role of macrophages/monocyte in ADCP and their FcγR has been comprehensively reviewed by Gorgon *et al* [84]. Studies with human purified T cells and NK cells from human peripheral blood in culture [6,11] or mixed lymphocyte reaction assay on human PBMC that are devoid of neutrophils showed FcγRI and FcγRIII but not FcγRII involved in NK cell-mediated ADCC. However, studies using transgenic mice expressing human CD2 confirmed depletion of T cells *in vivo* but NK cell depletion via anti-asialo GMl pretreatment did not inhibit alefacept-mediated T cell depletion in these mice. These data suggest that other cells expressing either FcγRI/FcγRIII might deplete T cells *in vivo*.

There are no studies that reported alefacept-mediated depletion of T cells *in vivo* via monocytes/macrophages in humans in addition to NK cells. Based on current understanding, we believe that alefacept might cause ADCP via monocyte/macrophages and might activate these cells for secretion of cytokines but this remains to be elucidated. How activation of FcγRI/FcγRIII^+^ accessory cells affect the physiology of HIV infected individual remains to be observed. Furthermore, preservation of primary or secondary antibody responses to either recall (tetanus toxoid) or a novel (ΦX174) antigen [76] indicates that immune response are intact and plausibly both APC and T cell function are not impaired due to alefacept treatment.

HIV patients also have a higher risk of malignancies, cardiovascular disease and other conditions presumably due to immune system dysregulation. Although alefacept has a good safety profile [85], the product insert poses several warnings including a potential increase in malignancy (http://www.accessdata.fda.gov/drugsatfda_docs/label/2012/125036s0144lbl.pdf). The incidence of malignancies was 1.3% (11/876) for alefacept-treated patients compared to 0.5% (2/413) in the placebo group over a 24-week period. Most of the malignancies (all except three; non-Hodgkin’s follicle-center cell lymphoma and Hodgkin’s disease) were non-melanoma skin cancers that are easily curable. A detailed description about these malignancies from the FDA website (http://www.fda.gov/downloads/Drugs/DevelopmentApprovalProcess/HowDrugsareDevelopedandApproved/ApprovalApplications/TherapeuticBiologicApplications/ucm086012.pdf) indicates that most of these cancers’ relationship to alefacept was classified as ‘none’. Regarding the three cases of lymphomas, except one (non-Hodgkin’s lymphoma) the relationship with alefacept was described as “likely” but unlikely from immunosuppression. These three subjects received more than 15 standard doses of alefacept. In view of this limited information and the known increased malignancy risk in HIV patients, whether alefacept will pose an additional risk to HIV patient is difficult to assess at this point.

Progressive multifocal leukoencephalopathy (PML), a rare, potentially fatal demyelinating disease, affects primarily immunocompromised individuals. Another T cell modulating agent, efalizumab, that targets multiple stages of T cell activation by interacting with CD11a, has been voluntarily withdrawn from the US market because of the risk of PML [86,87]. The question whether alefacept that is also a T cells modulating agent will introduce a risk to HIV infected individuals for PML during treatment remains to be seen. Currently, there are no reports of PML associated with alefacept since the 2002 FDA approval. This history of alefacept suggests that the risk is less likely.

The potential risks versus benefits of alefacept must be carefully measured similarly to any other HIV cure strategy. Once the beneficial impact of alefacept on eliminating or reducing the HIV latent reservoir can be confirmed, the risk vs. benefit data can be more thoughtfully considered.

## Current Therapeutics under Development for HIV Cure

Patients on ART for years without interruption stabilize their CD4^+^ T cell counts to near normal levels. However, these patients sustain chronic inflammation [88], drug side effects [89] and possible development of drug resistance. Therefore, HIV cure strategies currently being evaluated are intended to achieve either sterilizing cure, ART-free remission or functional cure. We will not review this in detail here as this has already been reviewed extensively [90,92–95,96,98,103,104]. Instead we will provide a brief overview to assist the reader in thinking about the potential utility of alefacept in the context of other broad categories of HIV cure strategies.

### Gene therapy

Post-transcriptional and transcriptional gene silencing of HIV-1 by using more than one non-coding RNAs (si/shRNAs) is being tested in phase I/II clinical trials. Some of these studies that completed phase II trials demonstrated that cell-modified gene therapy was safe and biologically active, with no adverse events but unable to reduce viral load. This approach focuses on targets that are highly conserved within the majority of HIV strains. Some examples are: (a) tat-vpr-specific anti-HIV ribozyme, termed OZ1, delivered in autologous CD34^+^ HPSCs [91]; (b) three anti-HIV-1 shRNAs, targeting highly conserved regions of the HIV integrase, protease and tat-rev genes, delivered in a single LV construct termed R3A [92]; (c) shRNA targeting the ccr5 gene and the C46 peptide fusion inhibitor in a LV construct termed LV sh5/C46 (or Cal-1), developed by Calimmune Inc.; and (d) shPromA and sh143 in a single therapeutic. This has been reviewed in reference [93].

The most promising study so far within the category of gene therapy comes from Sangamo Biosciences, where infusion of engineered CD4^+^ T cells (that carries deletion of CCR5 obtained by zinc finger nucleases) into HIV-1-infected individuals provided some control of HIV-1 replication *in vivo* [94]. This study is not yet complete and reviewed in [95].

LTR-directed Cas9/guide RNA (gRNA) demonstrated neither genotoxicity nor off-target editing to the host cells (microgial/monocytic cells). This strategy eradicated the HIV-1 genome and effectively immunizes target cells against HIV-1 reactivation and infection with high specificity and efficiency [96]. This method is promising but currently only demonstrated at the cellular level. It will take time to get to the clinics. Furthermore, strategies to deliver this genome editing technology *in vivo* have not yet been tested in human subjects.

### Vaccine

Development of a vaccine is one of the continuing efforts in HIV cure (therapeutic vaccine) and prevention (prophylactic vaccine). First, therapeutic T-cell based vaccines were developed in early 2000s but achieved, at best, modest results [97–100]. Next, effort was made to obtain broad neutralizing antibodies against the viral envelope. However, generation of these antibodies has proven extremely problematic due to the high degree of sequence variability in the envelope gene and the inaccessibility of stable epitopes [101]. Although various prophylactic vaccines for HIV have been tested in clinical trials, minimal success has resulted.

One preventative vaccine, known as RV144 for HIV clades B and E, tested in phase III clinical trials in Thailand, has shown some efficacy at preventing infection when compared with infection rates in a control group [102]. Progress toward a more effective vaccine development is underway.

Related to vaccine based strategies, a Phase I clinical trial of passive immunization with a large quantity of highly effective broad neutralizing antibody (bNAb), recently isolated from HIV infected patients, was well tolerated [103,104]. These bNAbs also has the potential to reduce a patient’s viral load without the use of ART.

### Shock and kill

The shock and kill strategy for HIV eradication is based on the concept that reactivation of HIV from latency will result in death of infected cells under the cover of ART. This may occur by viral cytopathic effect (CPE) and/or by the immune response. Latently infected cells, do not actively express genes. However, latency reversal agents (LRAs) reinitiate HIV viral antigen expression and then may solicit immune response to assist for the “kill” in addition to viral CPE. To date, no LRA has produced a substantial decrease in the size of the latent HIV reservoir. The reason might be due to a limited ability of CD8^+^ T cells of most HIV patients on ART to kill infected cells after reversal of latency, incomplete control of HIV by ART in anatomically protected viral sanctuaries and/or inadequate latency reversal in addition to many other possibilities [105,106].

All of these current approaches for achieving HIV cure are very innovative and not mutually exclusive. The strategies bear exciting promise but require more time and resources to be proven.

## Conclusion

The success of ART in controlling HIV infection and improving quality of life and reducing mortality rates is tremendous. However, viral rebound within two weeks after interruption of ART poses an absolute need for continuous use of ART. Thus, there is a great necessity to find a cure of HIV to reduce both the global economic burden as well as individual toxicity associated with ART. There are several innovative efforts underway including gene therapy, “shock and kill” approaches, improvement in ART and development of therapeutic vaccines for HIV cure. All of these strategies will require significant time, as their efficacy is still very uncertain.

Alefacept, since FDA approval in 2002, demonstrated an excellent safety profile and the expected results of selective depletion of memory T cell but not naïve T cells. Alefacept alone might be very useful in reducing the memory CD4^+^ T cell reservoir to achieve longer ART-free remission. It is plausible that in future, alefacept could be used in combinatorial strategies for more effective removal of latent HIV reservoir including cells other than memory CD4^+^ T cell.

Advantages discussed here of using alefacept as a therapeutic agent provide a strong rationale to repurpose this biologic to decrease the size of the latent HIV reservoir and concomitantly increase the virus- and ART- free life span of HIV infected individuals.
